# Spinal meningioma: clinical profile and outcome of surgical management

**DOI:** 10.11604/pamj.2022.43.44.19808

**Published:** 2022-09-27

**Authors:** Toyin Ayofe Oyemolade, Augustine Abiodun Adeolu, Adefolarin Obanisola Malomo, Matthew Temitayo Shokunbi, Ayodeji Akeem Salami

**Affiliations:** 1 Department of Neurological Surgery, University College Hospital, Ibadan, Nigeria,; 2 Department of Surgery, College of Medicine, University of Ibadan/University College Hospital, Ibadan, Nigeria,; 3 Department of Pathology, College of Medicine, University of Ibadan/University College Hospital, Ibadan, Nigeria

**Keywords:** Spinal meningioma, Nigerian, surgery, outcome

## Abstract

Spinal meningiomas are relatively rare tumours with often favourable surgical outcomes. There is paucity of data on spinal meningiomas in the Nigerian literature. This study was designed to evaluate the incidence, the anatomical distribution and outcome of surgical treatment of spinal meningiomas in our center. This was a retrospective study of patients who had surgery for spinal meningioma at our center during the study period. We obtain data from case note, operation and pathology registers. Univariate analysis was performed using SPSS version 15 (SPSS Science Inc; Chicago, IL, USA). There were 11 patients in the study all of whom were females with age range of 26-65 years. All the patients had motor deficit at presentation. Four patients (36.4%) presented within 6 months of onset of symptoms while the duration of symptoms was more than a year in 5 patients (45.5%). The preoperative functional grading was Frankel A in 2 patients (18.2%), Frankel D in 1 patient (9.1%), Frankel B and C each in 4 patients (36.4%). The tumours were located in the thoracic region in six patients (54.5%), cervical region in 2 patients (18.2%) while the tumours were cervicothoracic in the other 3 patients (27.3%). All the patients had gross total tumour resection. Post-operative neurological improvement occurred in 7 patients (63.6%) while 4 patients (36.4%) remained neurologically the same. All the patients in this study were women. Gross total tumour resection was achieved in all the cases with satisfactory functional outcome.

## Introduction

Meningiomas are typically benign tumours and may be intracranial or spinal in location [1]. Spinal meningiomas are relatively rare accounting for 7.5-12.7% of all meningiomas and 20-45% of intradural spine tumours [2,3]. They are more common in the middle-aged women with 2-3 times higher incidence in women than men [4,5]. They are usually well-circumscribed, slow-growing tumours [4,6]. They may cause spinal cord and nerve root compression with resultant pain, and sensory, motor and autonomic disturbances [3]. The clinical presentation is a function of tumour location as well as the rate of tumour growth and neural tissue compression. Treatment is predominantly surgical [1,4,7]. The outcome of surgical intervention is usually favourable [1,4]. Progressive neural tissue compression can however result in permanent neurological deficits even after successful surgery [4]. Hence the importance of early diagnosis and prompt treatment cannot be overemphasized. There is a paucity of these cases in the Nigerian medical literature and thus the clinical features and outcome of surgery for spinal meningioma among the local population remain largely elusive. This study aims to evaluate the incidence, anatomical distribution and outcome of surgical treatment of spinal meningiomas in our centre.

## Methods

**Study area:** the study setting is the department of Neurological Surgery, University College Hospital Ibadan. The University College Hospital Ibadan is the pioneer teaching hospital in Nigeria. It is a 800-bed tertiary health facility located in Ibadan, Southwest Nigeria.

**Study design:** this is a retrospective descriptive analysis of the cohort of our operated spinal tumours with meningioma.

**Study population:** all patients with histologically confirmed spinal meningioma in our centre between January 2004 and February 2017 were included in the study.

**Data collection:** data were collected from hospital case notes, operations and pathology registers. We obtained information on the age and sex of the patients, duration of symptoms before presentation, presenting symptoms, anatomical location of the tumours, imaging findings, preoperative and postoperative Frankel grading, outcome of surgeries and duration of follow-up.

**Definition:** the outcome of care was classified as good (Frankel grades D and E) and poor (Frankel grades A, B, C).

**Analysis:** statistical analysis was done using SPSS version 15 (SPSS Science Inc; Chicago, IL, USA). We calculated simple frequencies and percentages for the qualitative variables and the mean for the quantitative variables. The Fisher´s exact test was used to determine the association between duration of symptoms, tumour histology, preoperative functional grade, spinal region of tumour location and outcome of treatment. A p-value less than 0.05 was considered significant.

**Ethical consideration:** the global ethical recommendations regarding confidentiality and patient-specific data protection were strictly adhered to.

## Results

Socio-demographic characteristics: There were 11 patients in the study all of whom were females with the age range of 26-65 years. The mean age at presentation was 43.7± 14.3 years. Three patients (27.3%) each were in the 20-29 and 40-49 age groups.

**Figure 1:**
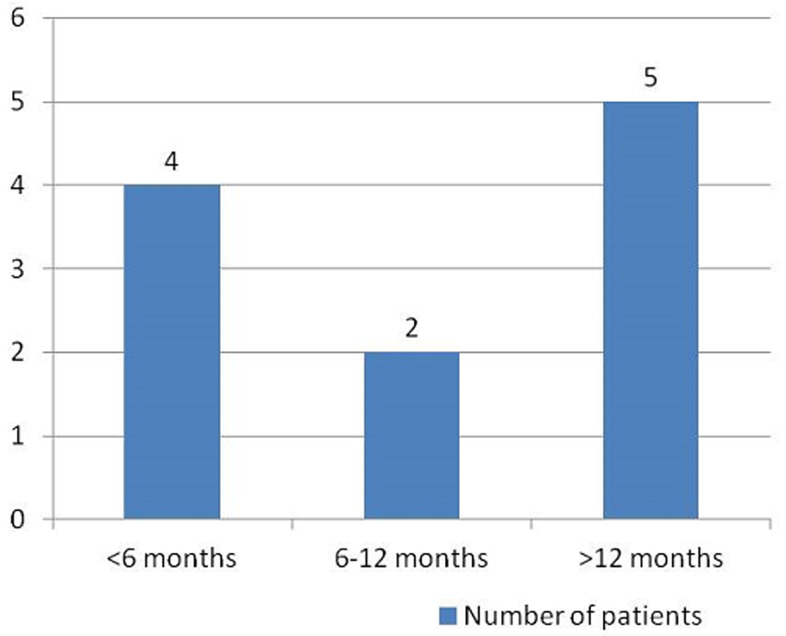
duration of symptoms at presentation

Clinical characteristics: All the patients had motor deficits at presentation while 72.7% had pain in addition to the motor deficit (Table 1). The duration of symptoms ranged from 2-60 months. Four patients (36.4%) presented within 6 months of the onset of symptoms while in 5 patients (45.5%), the duration of symptoms was more than a year (Figure 1). The preoperative functional grading was Frankel A in 2 patients (18.2%), Frankel D in 1 patient (9.1%), Frankel B and C each in 4 patients (36.4%) (Table 1). The tumours were located in the thoracic region in six patients (54.5%), a cervical region in 2 patients (18.2%) while the tumours were cervicothoracic in the other 3 patients (27.3%). All the tumours were located in the intradural-extramedullary compartment. All the patients had gross total tumour resection. The patients were followed up for a period of 4 to 24 months (mean= 10.27 months).

**Table 1:** demographic and clinical characteristics of the patients

SN	Age (years)	Sex	Clinical presentation	Duration of symptoms (months)	Spinal region	Histological type	Preop Frankel grading	Postop Frankel grading
1	48	F	Pain, Motor deficit	4	Thoracic	Psammomatous	D	D
2	51	F	Motor deficit	24	Cervical	Angiomatous	B	D
3	26	F	Pain, Motor deficit	2	Cervico-thoracic	Fibroblastic	C	C
4	30	F	Pain, Motor deficit	12	Thoracic	Psammomatous	A	E
5	65	F	Motor deficit	36	Cervical	Psammomatous	C	D
6	43	F	Motor deficit	12	Thoracic	Meningothelia	B	D
7	62	F	Pain, Motor deficit	18	Cervico-thoracic	Transitional	C	E
8	28	F	Pain, Motor deficit	5	Thoracic	Transitional	A	A
9	55	F	Pain, Motor deficit	14	Cervico-thoracic	Meningothelia	B	B
10	28	F	Pain, Motor deficit	60	Thoracic	Fibroblastic	B	D
11	45	F	Pain, Motor deficit	5	Thoracic	Meningothelia	C	D

**Figure 2:**
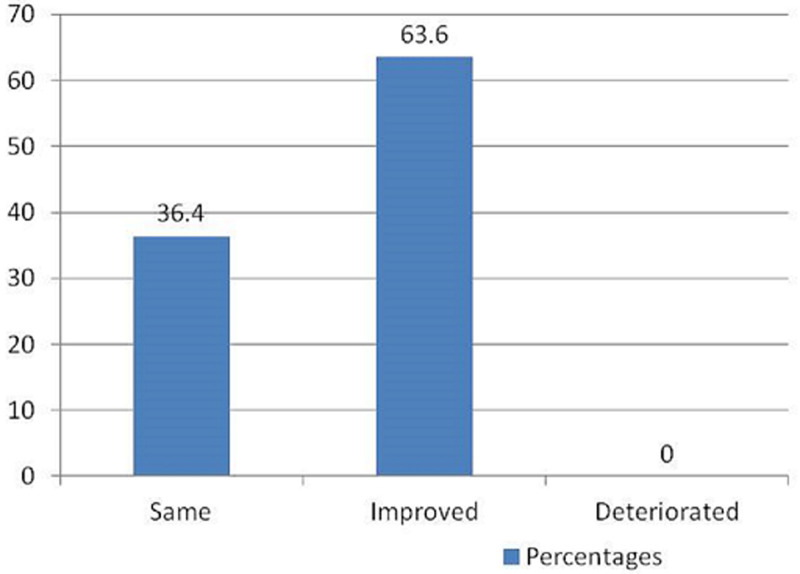
post-operative neurological outcome of the patients

Outcome: Post-operative neurological improvement occurred in 7 patients (63.6%) while 4 patients (36.4%) remained neurologically the same (Figure 2). The outcome of care was good in 8 patients (72.7%) and poor in 3 patients (27.3%). One patient had a superficial surgical site infection. There was no correlation between duration of symptoms (p= 0.382), spinal region of tumour location (p= 0.179), tumour histology (p= 0.627), preoperative functional status of the patients (p= 0.821) and outcome of management (Table 2).

**Table 2:** predictors of outcome of treatment

Clinical variables	Fisher’s exact test P value
Pre-operative neurological status	1.000
Tumour histology	0.782
Spinal region of tumour location	0.206
Duration of symptoms	0.515

## Discussion

Spinal meningiomas are relatively rare when compared to meningiomas in the intracranial compartment accounting for approximately 7.5-12.7% of all meningiomas [2,6]. They are usually benign, slow growing and respond favourably to surgical excision [8,9]. We evaluated eleven cases of spinal meningiomas operated in our centre over the study period. This number represents 12.64% of cases of spinal tumours operated in our centre during this period. All the patients in our study were females. The female predominance has been widely reported in the literature [1,2,10-12]. In the series by Mehrazin *et al*. 79% of the subjects were females [10]. The female population represents 58% and 79.6% in the series by Setzer *et al*. and Roux *et al*. respectively [1,11]. The female predominance is thought to arise from sex hormones or other receptor types common to women [13,14].

The reported peak incidence of spinal meningioma is in the 6th-8th decade of life [8,9,15]. The bulk of our patients was below this age range. Only 36.36% of our patients were in the sixth decade and above age range with 27.27% of the patient in the third and fifth decade each. The age range at presentation in this study was 26-65 years with a mean of 43.73±14.09 years. Iacob reported an age range of 34-72 years with a mean of 54.7 years, while Setzer reported a range of 20-91 and a mean of 61.9±16.0 years [1,7]. In the series by Arima *et al*,. the age range was 21-84 years and mean age was 60.3 years [16]. Spinal meningiomas often run an indolent course [1]. The delayed referral of patients with these tumours has been alluded to by Sandalcioglu *et al*. [6]. All our patients presented with motor weakness. This high incidence of motor weakness has been reported by other workers. Mehrazin *et al*. reported motor weakness in 92% of their patients while Sandalcioglu *et al*. 94% [6,10]. Setzer *et al*. however reported a 50% incidence of motor weakness in their series [1]. About 91% (10) of our patients were not walking at the time of presentation (Frankel grade A-C). This is higher than 39% reported in the series by Sandalcioglu *et al*. and 26.5% in the series by Klekamp *et al*. [6,17]. In the series by Roux *et al*., 66.6% of the patients were ambulatory at presentation [11].

**Figure 3:**
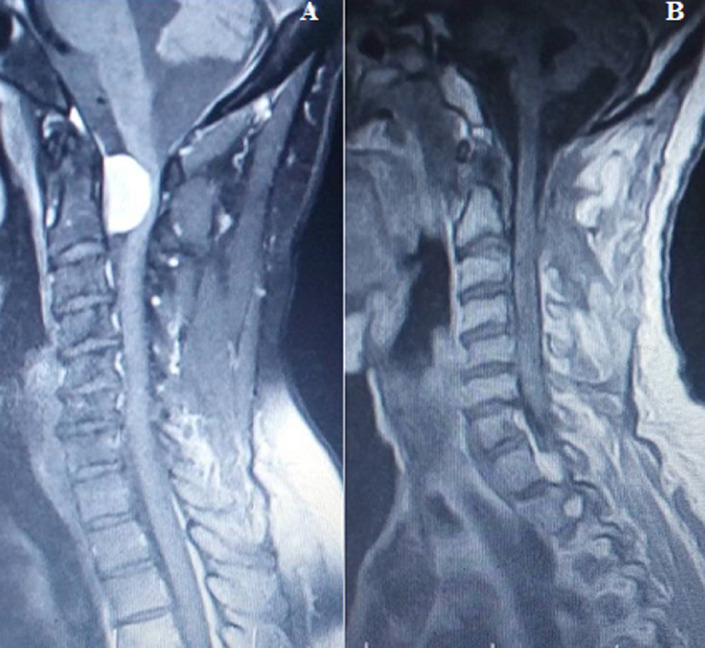
pre (A) and postoperative (B) cervical spine MRI images showing gross total tumour excision in one of our patients

The duration of symptoms in this study ranged between 2-60 months with a mean of 17.46 months. This mean is higher than the reported 13.7 months by Iacob, 11.8 months by Setzer *et al*. and 5.8 months by Mehrazin at al [1,7,10]. Five of our patients (45.5%) presented with symptoms of more than a year duration (Figure 1). This is similar to the 41% reported by Subaciute [4]. A larger proportion of our patients (36.4%) however presented within 6 months compared to the 9% in his series. In this present study, 18.2% of the tumours were located in the cervical region, 54.5% in the thoracic region and 27.3% traversed the cervical and thoracic spine. The thoracic predominance has been widely reported in the literature [1,4,10,12]. The thoracic location was seen in 60% in the series by Setzer *et al*., 78% in the work by Subaciute and 77.5% by Mehrazin *et al*. [1,4,10]. The incidence of cervical meningioma in our study correlates well with the reported 14-27% incidence [13]. The cervicothoracic location in our series (27.3%) is proportionally higher than 5% reported by Sandalcioglu *et al*., and 7.5% by Setzer *et al*. [1,6]. Location of spinal meningioma in the lumbar region is rare [6]. Meningioma in this location was seen in 1.5% of cases in the series by Sandalcioglu *et al*., 2.5% in the series by Mehrazin *et al*., and 3.8% in the series by Setzer *et al*. [1,6,10]. In our series lumber meningioma was not seen. Spinal meningioma are found mainly in the intradural-extramedullary compartment, however, a small percentage can be completely extradural or both extradural and intradural [2,12]. Intradural-intramedullary meningiomas are very rare but have been reported [18,19]. Intradural-extramedullary location of meningiomas accounted for 93% of the cases in the study by Riad *et al*, and 92% of the cases in the series by Subaciute [4,20]. In keeping with the literature, all the tumours in our series were located in the intradural-extramedullary compartment. Similar to the report by Mehrazin *et al*. and Riad *et al*. all our patients had gross total tumour excision [10,20] (Figure 3). High total tumour excision rate was also reported by Subaciute (92%) and Sandalcioglu *et al*. (97%) [4,6].

**Figure 4:**
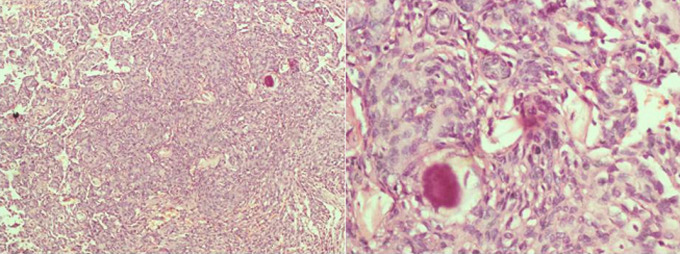
meningothelial meningioma (WHO grade 1) histological slides of patient 1

The functional outcome of surgical treatment of spinal meningiomas is generally favourable [1,6]. In our series 63.6% of the patients improved while 36.4% remained neurologically the same (Figure 2). Similar to the report by Setzer *et al*., there was no post-operative neurological deterioration in our series [1]. Eighty percent of the patients in the series by Mehrazin *et al*. and 96.2% of the cases in the series by Sandalcioglu *et al*. improved or remained neurologically the same [6,10]. Riad *et al*. reported neurological improvement in 87% of their cases [20]. In our study, 70% of the patient who was not ambulating at the time of presentation improved post-operatively. At the time of the last follow-up (24 months), one of the two paraplegic patients with preoperative duration of symptoms of 12 months had attained Frankel E functional status. All tumours in this study were WHO grade 1 in keeping with the reported predominance of WHO grade 1 meningioma in this location [12] (Figure 4). The post-operative outcome in our study was unaffected by the preoperative neurological status, preoperative duration of symptoms and histological subtype of the tumours (Table 2). The patients in our series were followed for a mean period of 10.27 months. The mean duration of follow-up of the patients that remained neurologically the same after surgery was however lower (8.54 months) than the group mean suggesting a possible better outcome than the reported percentages over a longer duration of follow-up. The lack of correlation between histological subtypes and functional outcome has been reported in the literature [12]. There is controversy in the literature about the role of the severity of the preoperative functional grading in predicting postoperative outcomes. Haegelen *et al*. and King *et al*. reported significant post-operative improvement in their patients with severe preoperative neurological deficits, while severe pre-operative neurological deficit was reported as a predictor of poor postoperative functional outcome by Gezen *et al*. and Saraceni *et al*. [8,15,21,22]. The reported morbidity and mortality rates following surgical management of spinal meningiomas are low. One of the patients in our study (9.1%) had a superficial surgical site infection representing the only complication in this series. There was no mortality in this study.

**Limitations:** the limitations of this study include a small sample size and a short duration of follow-up.

## Conclusion

Spinal meningiomas are mostly benign and slow-growing tumours. They represent 12.64% of the spinal tumours operated on in our facility during the study period. The tumours were mostly located in the thoracic region and intradural- extramedullary. All the cases were seen in women. Gross total tumour resection was achieved in all the cases and the functional outcome was satisfactory with all the patients either improving or remaining neurologically the same at the time of the last follow-up.

### What is known about this topic

- Spinal meningiomas are relatively rare;
- Progressive neural tissue compression by these tumors can result in permanent neurological deficits even after successful surgery;
- There is paucity of data on spinal tumours in sub-Saharan Africa.

### What this study adds

- Data on the clinical and histological profile of spinal meningiomas in a sub-Sahara African nation;
- Even in a resource challenged setting, the outcome of surgical management of spinal meningiomas is favourable.
